# Item

**Published:** 1903-06

**Authors:** 


### ITEM.

THE Mellier Drug Company, of St. Louis, has issued another beautiful art plaque, this one representing music. It is a panel-shaped picture, drawn in relief, the colors and white work giving it great harmony of effect. It conforms to the usual classic figures in form and pose, and is one of the most pleasing souvenirs that has been sent out to the profession.

